# Cardioplexol, a new cardioplegic solution for elective CABG

**DOI:** 10.1186/1749-8090-8-S1-P120

**Published:** 2013-09-11

**Authors:** K Kairet, J Deen, L Vernieuwe, A de Bruyn, S Kalantary, I Rodrigus

**Affiliations:** 1 University of Antwerp, Belgium; 2 Department of Cardiac Surgery, Antwerp University Hospital, Belgium

## Background

Cardioplexol® (Swiss Cardio Technologies) is a hyperkalemic, low-dose, single-shot cardioplegic solution which offers immediate asystole of the heart, maintenance up to 90 minutes and immediate reversal of asystole after release of cross clamp. Intermittent crossclamping with Lidoflazine is the current operative technique used in our center. Main question was whether Cardioplexol is an efficient, safe, easy-to-use and worthy alternative to Lidoflazine in elective isolated CABG in low-risk patients. Primary outcomes are heart enzymes (cTnI and CK-MB) and secondary outcomes are operation times, length of stay, major complications and in-hospital mortality.

## Methods

From December 2011 to May 2013 40 patients, with LVEF ≥50%, EuroSCORE II <3,5% and no severe systemic disease, underwent elective CABG and were consecutively randomized to intermittent crossclamping (ICC; n=20) or Cardioplexol (CPX; n=20). All were operated on by the same surgeon.

## Results

There was no significant difference in age (CPX 71.04±8.54; ICC 67.25±9.90), EuroSCORE II (CPX 1.19±0.63; ICC 1.11±0.46) or number of distal anastomoses (CPX 2.95±0.51; ICC 3.15±0.745). We found no significant difference for maximum cTnI (CPX 3.38±1.50; ICC 4.59±4.23) or maximum CK-MB (CPX 23.99±13.95; ICC 25.50±20.10). ECC time (CPX 57.55±11.93; ICC 67.20±21.78) and cross-clamp time (CPX 24.62±1.31; Lido 22.76±1.49) were not significantly different. Neither length of hospital stay (CPX 9.10±2.32; ICC 8. 19±1,25) nor postoperative complications (CPX 1.25±0.78; ICC 0.85±0.67) showed significant difference. There was no in-hospital mortality.

## Conclusion

In this elective CABG population with low risk, Cardioplexol offered good myocardial protection, with comparable primary and secondary outcomes. Further studies are needed to expand its use.

